# Pregnancy and neonatal outcomes in Eastern Democratic Republic of the Congo: a systematic review

**DOI:** 10.3389/fgwh.2024.1412403

**Published:** 2024-12-05

**Authors:** Kambale Kasonia, Hannah Brindle, Daniela Manno, Tansy Edwards, Soazic Gardais, Grace Mambula, Zephirin Mossoko, Edward M. Choi, Nicholas E. Connor, Pierre Mukadi, Rebecca F. Grais, Babajide Keshinro, Chrissy H. Roberts, Hugo Kavunga-Membo, Daniel G. Bausch, Jean-Jacques Muyembe, Deborah Watson-Jones

**Affiliations:** ^1^ Clinical Research Department, Faculty of Infectious and Tropical Diseases, London School of Hygiene and Tropical Medicine, London, United Kingdom; ^2^ MRC International Statistics and Epidemiology Group, Faculty of Epidemiology and Population Health, London School of Hygiene & Tropical Medicine, London, United Kingdom; ^3^ School of Tropical Medicine and Global Health, Nagasaki University, Nagasaki, Japan; ^4^ Research Department, Epicentre, Paris, France; ^5^ Institut National de Recherche Biomédicale, Kinshasa, Democratic Republic of the Congo; ^6^ Janssen Vaccines and Prevention B.V., Leiden, Netherlands; ^7^ Department of Disease Control, Faculty of Infectious and Tropical Diseases, London School of Hygiene & Tropical Medicine, London, United Kingdom; ^8^ FIND, Geneva, Switzerland; ^9^ Mwanza Intervention Trials Unit, National Institute for Medical Research, Mwanza, Tanzania

**Keywords:** pregnancy, neonatal, outcomes, eastern, DRC, conflict

## Abstract

**Background:**

Conflict is known to impact maternal and neonatal health in Eastern Democratic Republic of the Congo (DRC), an area of longstanding insecurity. We conducted a systematic review on pregnancy and neonatal outcomes in this region to provide a comprehensive overview of maternal and neonatal outcomes over a 20-year period.

**Methods:**

We systematically searched databases, such as Medline, EMBASE, Global Health, ClinicalTrials.gov and the Cochrane Library, along with grey literature, for articles published between 2001 and 2021. These articles provided quantitative data on selected pregnancy and neonatal outcomes in the provinces of Ituri, Maniema and North and South Kivu, Eastern DRC. We conducted a descriptive analysis, combining results from different data sources and comparing incidence of outcomes in North Kivu with those in other provinces in Eastern DRC.

**Results:**

A total of 1,065 abstracts from peer-reviewed publications and 196 articles from the grey literature were screened, resulting in the inclusion of 14 scientific articles in the review. The most frequently reported pregnancy complications were caesarean sections (11.6%–48.3% of deliveries) and miscarriage (1.2%–30.0% of deliveries). The most common neonatal outcomes were low birth weight (3.8%–21.9% of live births), preterm birth (0.9%–74.0%) and neonatal death (0.2%–43.3%).

**Conclusion:**

Our review provides data on pregnancy and neonatal outcomes in Eastern DRC, which will be valuable for future studies. Despite the area's ongoing armed conflict, the percentages of complications we noted in Eastern DRC are comparable with those observed in other countries in the region.

**Systematic Review Registration:**

https://www.crd.york.ac.uk/PROSPERO/display_record.php?RecordID=262553, PROSPERO (CRD42021262553).

## 1 Introduction

Armed conflict has been shown globally to negatively impact maternal and child health often through indirect means such as reduced vaccination coverage or limited access to healthcare, including antenatal care and skilled birth attendants (1–4). The Eastern Democratic Republic of the Congo (DRC) has a long history of conflict, with wars from 1996 to 2003 followed by continued insecurity due to the presence of numerous armed rebel groups and non-state actors (5), some of which operate in the areas next to Goma (6). As a result, humanitarian organisations have been present in Eastern DRC for many years (5), with many providing maternal and child healthcare (7–14). This influx of aid, potentially led to claims that the health system in Eastern DRC was the best in the country (15), suggesting a more complex situation compared to the typically negative impact of conflict on maternal and child health reported in other countries. In 2016, Lindskog found no association between neonatal mortality and conflict in Eastern DRC (16), Kandala et al. (2014) reported that the under-five mortality rate in DRC was lowest in North Kivu (17) and Guo et al.(2021) observed that the adjusted coverage of antenatal care and skilled birth attendance increased in North Kivu and South Kivu (18). Additionally, due to the high prevalence of gender-based and sexual violence in the region and the subsequent occurrence of gynaecological fistulas, many obstetric services have been implemented to address these complications (19–21). These services may indirectly benefit maternal and child health.

Although previous studies have provided data on these outcomes in this conflict-affected area, a comprehensive review of the literature will be helpful to determine what rates of adverse maternal and neonatal outcomes have been documented to allow monitoring of trends over time as the conflict continues.

## 2 Materials and methods

### 2.1 Scientific articles

#### 2.1.1 Search strategy

One researcher (HB) extracted abstracts from Medline, EMBASE, Global Health, ClinicalTrials.gov (clinicaltrials.gov) and the Cochrane Library (cochranelibrary.com) databases on 27 May 2021 for scientific articles using the search terms and limits provided in Supplementary Material S1. Duplicate abstracts were removed.

One reviewer (HB) searched the English-language grey literature on 5 July 2021, and a second reviewer (KK) searched the French-language grey literature on 7 July 2021. Searches were performed using the Google search engine, the websites for the DRC Demographic Health Survey Program (DHS) (dhsprogram.com), the ReliefWeb (reliefweb.int), the MedRxiv website (medrxiv.org), the bioRxiv (biorxiv.org) website, and the websites of non-governmental organisations (NGOs) working in the region, including the United Nations International Emergency Children's Fund (UNICEF; unicef.org), Médecins Sans Frontières (MSF; msf.org), and the United States Agency for International Development (USAID; usaid.gov) using the search terms provided in Supplementary Material S2.

#### 2.1.2 Screening

At least two reviewers (including KK, HB, SG, GM, ZM, DM) independently screened abstracts from the database search, or full articles from the grey literature search.

Scientific articles or grey literature were included if they reported pregnancy outcomes such as miscarriage, pre-eclampsia, eclampsia, gestational diabetes, hyperemesis gravidarum, maternal anaemia, intrauterine growth restriction (IUGR), placenta praevia, maternal death, stillbirth, birth outcomes in caesarean section and reasons for caesarean section, preterm rupture of membranes, post-partum haemorrhage or maternal death, or neonatal outcomes, such as preterm birth, low birth weight, neonatal death, congenital anomalies, Apgar score, small for gestational age or prolonged hospitalisation; were in English or French; conducted in Eastern DRC (i.e., Ituri, North Kivu, South Kivu or Maniema provinces); were published between 2001 and 2021; were observational or interventional studies; and for grey literature, were published by either the DRC government (including DHS surveys) or by ReliefWeb, UNICEF, MSF or USAID. Case series, case studies, scientific articles and grey literature which only included qualitative results were excluded.

We used a checklist (Supplementary Material S3) to screen the abstracts and articles. Each reviewer gave a final recommendation as “include”, “exclude” or “unsure”. In the event of discordant views, a third independent reviewer was added and, if they agreed with one of the preceding two reviewers, that was considered the final decision. If there was no concordant decision (e.g., “include”- “exclude”- “unsure”), a fourth reviewer was added, with the final decision based on the majority recommendation.

If both initial reviewers classified an abstract as “unsure”, the full text was screened. For studies identified on ClinicalTrials.gov for which the classification was “unsure” or “include”, a single reviewer screened any linked publications from that study. To ensure that we did not miss any publication, we screened reference lists from each of the articles included in the review based on the title. Those articles that had not been screened were added to the full screening process.

**Figure 1:**
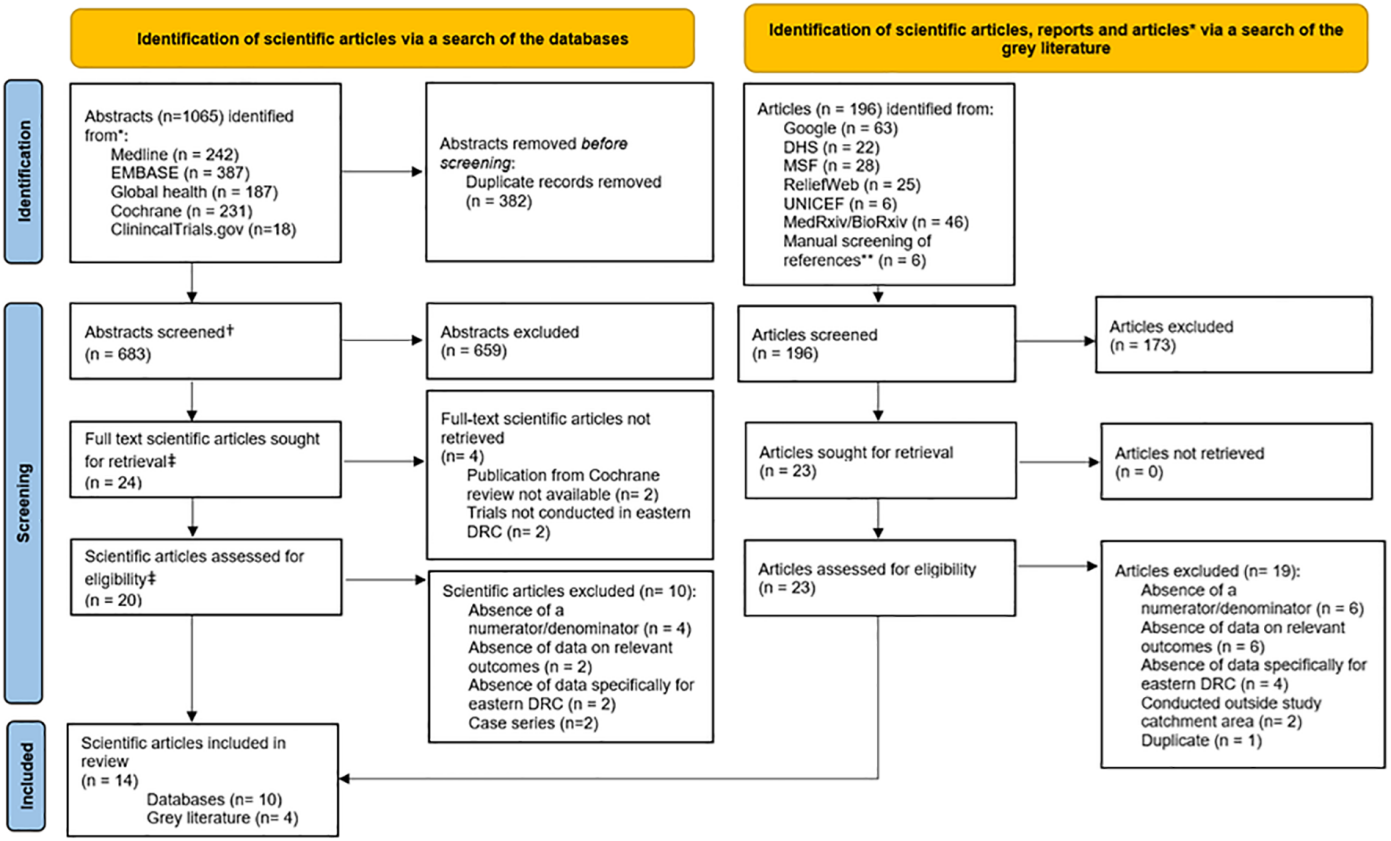
PRISMA flow diagram of article selection. * Referred to as ‘articles’. ^†^ For ClinicalTrials.gov, this refers to the study details published online. ** Previously screened references were excluded. ^‡^ For ClinicalTrials.gov, this refers to the number of trials. We used PRISMA flow diagram template available from https://www.prisma-statement.org/prisma-2020-flow-diagram.

For peer-reviewed scientific articles, we identified a total of 1,065 relevant abstracts, including 242 from Medline, 387 from EMBASE, 187 from Global Health, 231 from Cochrane and 18 from ClinicalTrials.gov. We removed 382 duplicate records, leaving 683 abstracts to be screened. Of these, 20 articles were eligible for data extraction. For grey literature, we identified 196 articles and reports, including 63 via a Google search, 22 from the DHS, 28 from the MSF website, 25 from the ReliefWeb website, six from the UNICEF website and 46 from the MedRxiv/BioRxiv preprint websites. We also identified six records for screening from references in publications assessed for eligibility. This process resulted in 23 eligible grey literature articles for data extraction (Figure 1).

Of the total of 43 articles which were eligible from the database search and from the grey literature search, 29 were excluded because numerators and/or denominators were absent (*n* = 10), the articles did not include data or outcomes relevant to our review (*n* = 8), there were no data from Eastern DRC (*n* = 6), the articles were case series (*n* = 2), the studies were conducted outside the defined geographical area for our review (*n* = 2) or the article was a duplicate (*n* = 1) (Figure 1 and Supplementary Material S4). In total, 14 publications were included in this systematic review, comprising ten from the database search and four from the grey literature search.

#### 2.1.3 Data extraction and quality assessment

We obtained the full text of all scientific articles to be included in the review and at least two independent reviewers (including HB, DM, KK, and ZM) extracted data using a form created for that purpose (Supplementary Material S5), while checking for consistency between reviewers. Scientific articles that were not open access were obtained from the London School of Hygiene and Tropical Medicine (LSHTM) library or through direct contacts with the author. Data from reports (for example, those published from the DHS) and articles (for example, those published by NGOs) were extracted by a single reviewer. We assessed the validity of scientific articles using the National Institutes of Health National Heart, Lung and Blood Institute Study Quality Assessment Tools for observational cohorts and cross-sectional studies (22). At least two reviewers independently scored the articles, with the inclusion of a third or a fourth reviewer in the event of discordant results. Each reviewer provided an overall assessment of “good”, “fair” or “poor” based on the quality of the publication, with the majority grade presented.

**Figure 2:**
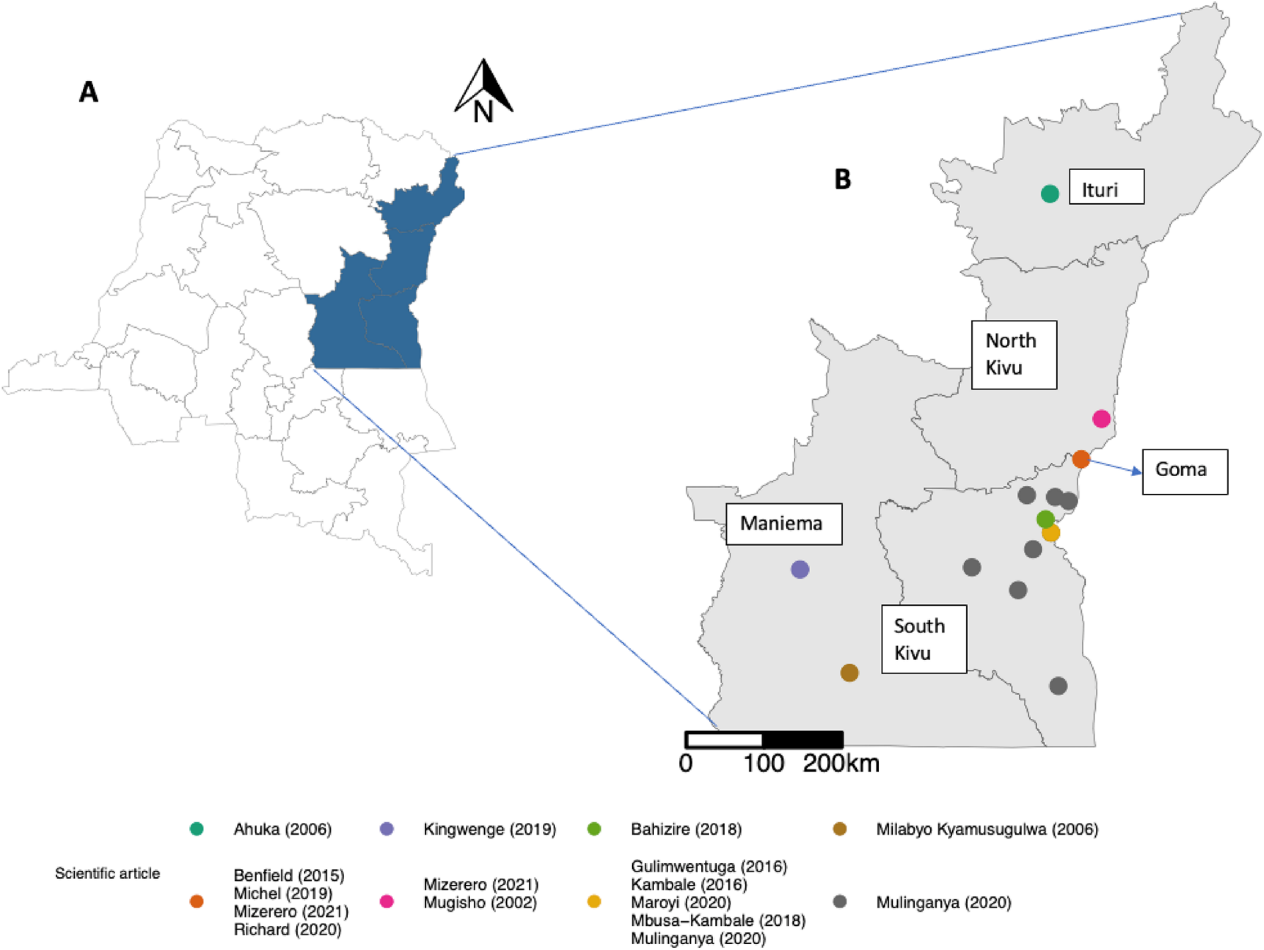
Geographic distribution of the study sites in the included scientific articles. The areas shaded blue in **(A)** are the provinces included in the review. The coloured dots in **(B)** represent the GPS coordinates of the scientific articles. The map was created using R software. Coordinates for the research sites of the different publications came from Google. The shapefile (base layer) for making the map came from Humanitarian Data Exchange. https://data.humdata.org/dataset/cod-ab-cod.

A summary table of the 14 final articles identified for analysis, including the pregnancy and neonatal outcomes provided, whether these were included in our analysis according to the inclusion criteria, and the final grades from the quality assessment are given in Table 1. Figure 2 shows the geographic distribution of the study sites in these articles, created using R statistical software (37) with the GPS coordinates of these sites obtained from Google and the Shapefile (baseline) from Humanitarian Data Exchange (38).

**Table 1:** Summary and quality assessment of scientific articles included in the analysis.

Number (reference)	First author, year	Region	Study design and setting	Study population	Outcomes	Included in our analysis	Quality
1 (23)	Ahuka, 2006	Irumu, Ituri	Retrospective cross-sectional study, *Centre Médical Evangélique*, a 250-bed referral hospital in Nyankunde, Ituri subregion, Oriental Province. The 30-bed maternity unit served as the referral hospital for high-risk pregnancies in Ituri.	8,824 babies delivered alive at the maternity unit from January 1993 to August 2001	Congenital anomalies	Yes	Fair
2 (24)	Bahizire, 2018	Miti-Murhesa, South Kivu	Prospective cohort study nested within a study evaluating the effectiveness of intermittent preventive treatment with Sulfadoxine-Pyrimethamine during pregnancy (IPTp-SP) conducted in health facilities of Lwiro, Miti-Murhesa Health zone, South Kivu Province.	478 pregnant women (residents of Miti-Murhesa Health Zone) recruited during their second trimester (12 to 24 weeks) between November 2010 and July 2011 at their first antenatal visit at Lwiro (*n* = 370) and Murhesa health facilities (*n* = 108). 355 single full-term live neonates	Low birth weight	No	Good
					Miscarriage	Yes	
					Maternal anaemia	No	
					Maternal death	No	
					Preterm birth	No	
					Stillbirth	Yes	
3 (25)	Benfield, 2015	Goma, North Kivu	Retrospective cross-sectional study to examine risk factors for fistula development, conducted in HEAL Africa Hospital, Goma, North Kivu Province. This tertiary hospital is owned and managed by HEAL Africa, an NGO. and a specific fistula care programme	187 women from Eastern DRC who underwent fistula surgery between 1 April 2009 and 1 March 2012 and who were asked about previous caesarean sections (North Kivu *n* = 121, South Kivu *n* = 18, Maniema *n* = 48), Women from other provinces not included in review.	Caesarean section	Yes	Fair
					Neonatal death	Yes	
					Stillbirth	Yes	
4 (26)	Gulimwentuga, 2016	Bukavu, South Kivu	Retrospective cohort study, Department of Surgery, *Hôpital Provincial Général de Référence* de Bukavu (HPGRB). This is a tertiary hospital in Bukavu, South Kivu.	30 neonates aged 1–30 days who were hospitalised at HPGRB from January 2010 to December 2013 for a surgical emergency (whether operated on or not).	Congenital anomalies	Yes	Poor
					Neonatal death	Yes	
					Preterm birth	No	
5 (27)	Kambale, 2016	Bukavu, South Kivu	Cross-sectional study conducted in the neonatology ward, Department of Paediatrics, HPGRB, South Kivu.	1,638 neonates aged 0–28 days with complete medical records hospitalised from January 2009 to December 2013.	Apgar score	Yes	Fair
					Congenital anomalies	Yes	
					Low birth weight	Yes	
					Neonatal death	Yes	
					Preterm birth	Yes	
					Prolonged hospitalisation	No	
6 (28)	Kingwenge, 2019	Kindu, Maniema	Cross-sectional study conducted in *l’Hôpital Général de Référence de Kindu* (HGRK), town of Kindu, Maniema Province.	1230 neonates admitted to the HGRK Neonatology Unit between 2017 and 2018.	Preterm birth	Yes	Fair
					Low birth weight	Yes	
					Neonatal death	Yes	
					Prolonged hospitalisation	No	
7 (29)	Maroyi, 2020	South Kivu	Cross-sectional study conducted in maternity wards of five tertiary care hospitals in South Kivu (Ifendula and Kalonge rural hospitals; Nyantende and Rau suburban hospitals and Panzi urban hospital).	422 women who delivered (vaginally or via planned or emergency caesarean section) with a history of two or more previous caesarean sections at one of the five tertiary care hospitals in South Kivu.	Caesarean section,	Yes	Good
					Reasons for caesarean section	Yes	
8 (30)	Mbusa-Kambale, 2018	Bukavu, South Kivu	Prospective cohort study with enrolment at the Neonatology Unit, Reference Provincial General Hospital of Bukavu (HPGRB) over a period of six months.	200 mother-child pairs (100 infants randomly selected from 183 low birth weight infants w (<2,500 g) and 100 infants randomly selected from 413 infants born at full-term with birth weight of ≥2,500 g Pair enrolled between January 2016 and December 2016.	Intrauterine growth restriction	Yes	Good
					Preterm birth	Yes	
					Prolonged hospitalisation	No	
9 (31)	Michel, 2019	Goma, North Kivu	Cross-sectional study in four referral hospitals in Goma, North Kivu (Virunga Hospital, Bethesda Hospital, *Charité Maternelle* Hospital and Goma Provincial Referral Hospital)	736 women who delivered by caesarean section between 1 November 2013 and 1 January 2016 at one of the four hospitals.	Miscarriage	Yes	Fair
					Placenta praevia	Yes	
					Maternal death	Yes	
					Stillbirth	Yes	
					caesarean section	Yes	
					Reasons for caesarean section	Yes	
					Low birth weight	Yes	
					Neonatal death	Yes	
					Apgar score	Yes	
10 (32)	Milabyo Kyamusugulwa *et al*., 2006	Kama and Kipaka, Maniema	Cross-sectional study, maternity wards of 2 rural health facilities: Kama Referral Health Centre and Kipaka General Referral Hospital Maniema Province.	938 livebirths born between November 2003 and October 2004	Low birth weight (<2,500 g)	Yes	Good
11 (33)	Mizerero, 2021	North Kivu	Cross-sectional study in maternal and neonatal health departments of 42 public health facilities including referral hospitals, referral health centres and health centres in Goma (*n* = 11), Karisimbi (*n* = 14) and Rutshuru (*n* = 17) Health Zones, North Kivu.	44,042 births in 2016			
					Miscarriage	Yes	Fair
					Pre-eclampsia	Yes	
					Maternal death	Yes	
					caesarean section	Yes	
					Post-partum haemorrhage	No	
					Neonatal death	Yes	
12 (34)	Mugisho, 2002	Rutshuru, North Kivu	Cross-sectional study, maternity ward, Rutshuru Referral Hospital, North Kivu.	13,042 women who delivered at Rutshuru Referral Hospital between 1980 and 1998. Refugees were excluded.	Caesarean section	Yes	Fair
					Maternal deaths	Yes	
13 (35)	Mulinganya, 2020	South Kivu	Cross-sectional study in 30 secondary health facilities (11 government-owned, 14 faith-based and 5 private) in eight health zones South Kivu	5,520 women who delivered by caesarean section in any of the 30 health facilities in 2018.	Miscarriage	Yes	Good
					Caesarean section	Yes	
					Reasons for caesarean section	No	
					Preterm birth	Yes	
					Low birth weight	Yes	
14 (36)	Richard, 2020	Goma, North Kivu	Case-control study conducted in seven hospitals (undefined) in Goma, North Kivu to examine the association between vitamin D status and risk of pre-eclampsia	Cases: 95 pregnant women diagnosed or followed up for pre-eclampsia. Controls: 95 women attending antenatal clinic visits in the same facility as a case who had normal pregnancies and no chronic/debilitating conditions.	Miscarriage	Yes	Good
					Pre-eclampsia	Yes	

### 2.2 Study registration and ethics

We registered the systematic review with PROSPERO (CRD42021262553) on 12 July 2021 (39). We made two amendments to the protocol following initial registration: (1) a change of the criteria used for the quality assessment and (2) a correction to the search criteria. Ethical approval was not required for this review.

### 2.3 Analysis

Pregnancy and neonatal outcomes in North Kivu and other provinces were analysed descriptively using the statistical software, R (37). We calculated the percentage and corresponding 95% confidence intervals (CI) of each of the outcomes based on numerators and denominators provided within each scientific article (Supplementary Material S6) using the R function “prop.test” and presented these using the R package “ggplot2” (40). Non-standard denominators were referred to as those which included a subset of the total number of deliveries or pregnant women.

## 3 Results

### 3.1 Pregnancy outcomes

Pregnancy outcomes were obtained from nine scientific articles including six cross- sectional studies two case-control studies and one prospective cohort study (24) (Table 1). Of the scientific articles, the quality was judged to be good in five (24, 30, 36) and fair in four (25, 31, 33, 34) (Table 1).

#### 3.1.1 Caesarean sections

**Figure 3:**
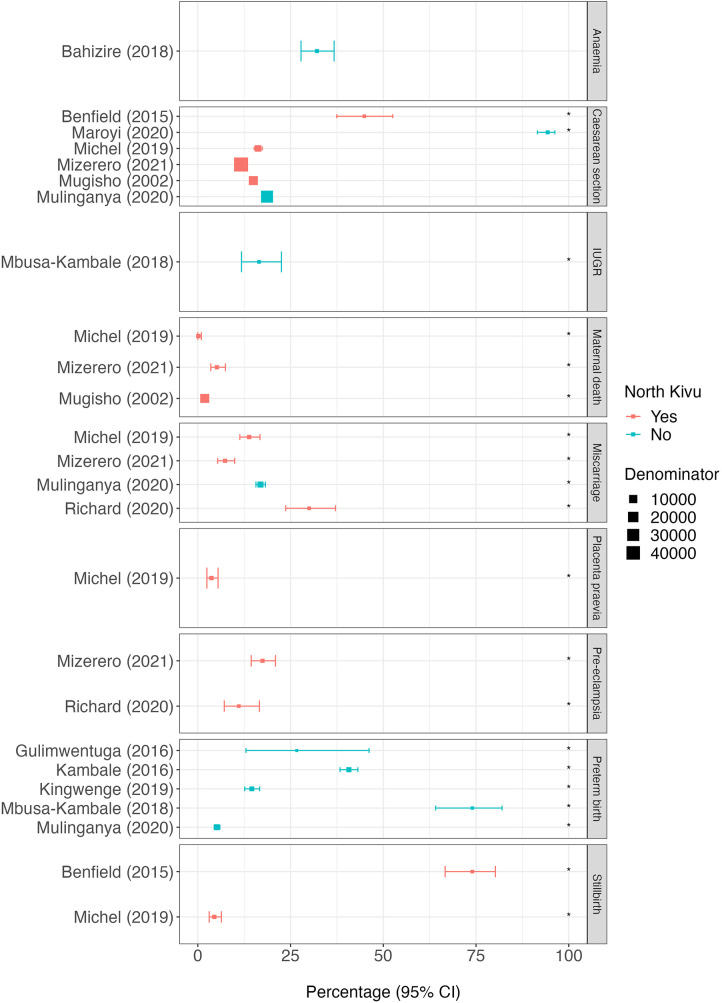
Percentage of pregnancy outcomes reported by each scientific article. The position of squares represents the percentage of subjects with each outcome and bars the 95% confidence interval (95%CI). The size of the squares is weighted accorded to the size of the denominator (provided in supplementary material 6). Red squares represent scientific articles conducted in North Kivu and blue squares conducted outside North Kivu. The asterisk (*) indicates scientific articles for which the denominator is non-standard. Placenta praevia in the study by Michel (2019) is given as the post-operative diagnosis only.

The percentage of caesarean sections was obtained from six facility-based cross-sectional studies (25, 29, 31, 33–35), of which two used a non-standard denominator (Table 1 and Figure 3). In North Kivu, of the scientific articles for which the denominator was deliveries in health facilities, the percentage of reported caesarean sections ranged from 11.7% (95%CI 11.4%–12.0%, *n* = 5134/44,042) (33) to 16.2% (95%CI 15.2%–17.4%, *n* = 736/4,530) (31). In the study which included only women who underwent fistula surgery, the percentage of women who had a caesarean section before the surgery was 44.9% (95%CI 37.5%–52.5%, *n* = 79/176) (25).

**Figure 4:**
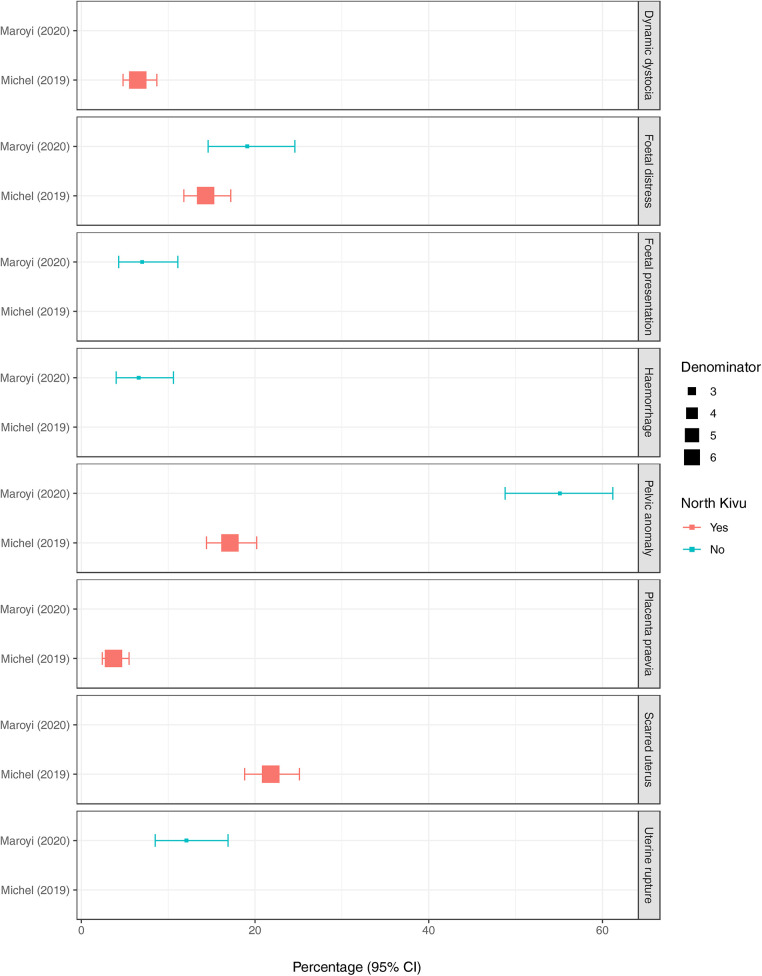
The position of squares represents the percentage of subjects with each outcome and bars the 95% confidence interval (95%CI). The size of the squares is weighted according to the size of the denominator (provided in supplementary material 6). Red squares represent scientific articles conducted in North Kivu and blue squares, conducted outside North Kivu. The indications in the scientific article by Michel (2019) are those provided as the post-operative diagnoses only.

Outside North Kivu, the percentage of caesarean sections among all hospital deliveries was 18.6% (95%CI 18.2%–19.1%, *n* = 5,520/29,600) (35) and among those with a history of previous caesarean section, the percentage of pregnant women experiencing another caesarean section was 94.3% (95%CI 91.5%–96.2%, *n* = 398/422) (29) (Figure 3). In North Kivu, the most likely indication for a caesarean section was a ’scarred uterus’ (21.8%, 95%CI 18.8%–25.1%, *n* = 148/680) (31) whereas in South Kivu it was “abnormality of the pelvis” (55.1%, 95%CI 48.8%–61.2%, *n* = 141/256) (29) (Figure 4).

#### 3.1.2 Maternal deaths

Data on the percentages of maternal deaths in North Kivu were available from three studies (one prospective cohort study and two cross-sectional studies) conducted in hospitals or referral facilities (31, 33, 34), all of which used non-standard denominators (Table 1 and Figure 3). Mugisho et al., 2002 reported that 1.9% (95%CI 1.6%–2.1%, *n* = 243/13,042) of all deliveries (excluding mothers who were refugees) ended in maternal deaths in Rutshuru hospital over an 18-year period (1980–1998). In a separate study conducted in 42 public health facilities in a single year (2016), among women who had an obstetric complication, 5.1% died (95%CI 3.5%–7.4%, *n* = 28/545) and in another cross-sectional study conducted in four referral hospitals in Goma, 0.1% (95%CI 0%–1.0%, *n* = 1/676) of women who had a caesarean section died (31).

#### 3.1.3 Miscarriage

Data on the percentage of pregnancies that ended in a miscarriage were obtained from three cross-sectional studies (two conducted in North Kivu and one in South Kivu) and one case-control study (in North Kivu) (31, 33, 35, 36), all of which used non-standard denominators (Table 1 and Figure 3). Of the studies conducted in North Kivu, the percentage of women who experienced a miscarriage ranged from 7.3% (95%CI 5.4%–9.9%, *n* = 40/545) among those with obstetric complications (33) to 13.8% (95%CI 11.3–16.8%) in the past medical history of those who had a caesarean section (31) to 30% (95%CI 23.7%–37.1%, *n* = 57/190) of those recruited into a case-control study in which half of the participants had pre-eclampsia (36). In South Kivu, one cross-sectional study reported a percentage of miscarriage of 16.9% (95%CI 15.7%–18.2%, *n* = 578/3,420) among pregnant women who had a caesarean section in a previous pregnancy (35) (Figure 3).

#### 3.1.4 Preterm births

The percentage of maternal deliveries resulting in preterm births was obtained from five scientific articles conducted outside North Kivu, including three cross-sectional studies, one case-control study (30), and one retrospective cohort study (26), all of which used non-standard denominators (Table 1 and Figure 3). The lowest percentage of preterm births was reported in the cross-sectional study conducted in 30 secondary health facilities in South Kivu, at 5.1% (95%CI 4.4%–6.0%, *n* = 168/3,272) among women who delivered by caesarean section (35). The other studies all included either hospitalised infants or those with low birth weight with the percentage of preterm births ranging from 14.6% (95%CI 12.7%–16.7%) to 74% (95%CI 64.1%–82%) (28, 30) (Figure 3).

#### 3.1.5 Pre-eclampsia and eclampsia

Of the scientific articles, the percentage of participants with pre-eclampsia was obtained from two studies conducted in North Kivu including the cross-sectional study conducted in 42 health facilities (33) and a case control study conducted in Goma (36), both of which used non-standard denominators (Table 1 and Figure 3). In the former, pre-eclampsia was diagnosed in 17.4% (95%CI 14.4%–20.9%, *n* = 95/545) of women with obstetric complications. In the latter, among the cases (who were diagnosed with or followed up for, pre-eclampsia) and controls (women attending antenatal care with normal pregnancies and no chronic/debilitating conditions), 11.1% (95%CI 7.1%–16.6%, *n* = 21/190) had a history of previous pre-eclampsia (36).

#### 3.1.6 Other outcomes

The percentages of women experiencing IUGR, maternal anaemia, stillbirth, and placenta praevia were obtained from four scientific articles including cross-sectional studies (25, 31), and two prospective cohort studies. None of these outcomes included scientific articles from both North and South Kivu.

In North Kivu, the percentage of stillbirth was obtained from two cross-sectional studies (25, 31), both of which used non-standard denominators (Table 1 and Figure 3). Among women who had caesarean section in four referral hospitals in Goma, the percentage who had a stillbirth was 4.4% (95%CI 3.1%–6.4%, *n* = 30/676) (31). In a study among the women who underwent fistula surgery, 74.0% (95%CI 66.7%–80.2%, *n* = 128/183) reported a stillbirth in their past medical history (25). The percentage of women with placenta praevia was obtained from one cross-sectional study in Goma, North Kivu where 7.4% (95%CI 5.6%–9.7%, *n* = 51/690) of those who had a caesarean section were diagnosed postoperatively (31).

The percentage of neonates who had IUGR was 16.5% (95%CI 11.8%–22.5%, *n* = 33/200) in a prospective cohort study in South Kivu whom half of the neonates had a birth weight of less than 2,500 g. In South Kivu, a prospective cohort of pregnant women recruited at their first antenatal visit reported maternal anaemia in 32.1% (95%CI 27.8%–36.7%, *n* = 141/439) (24) (Figure 3).

### 3.2 Neonatal outcomes

Eleven publications reported neonatal outcomes, including eight cross-sectional studies (23, 25, 27, 28, 31–33, 35), one prospective cohort study (24), one retrospective cohort study (26), and one case-control study (30). Of these, the quality was judged to be good in four (24, 30, 32, 35), fair in six (23, 25, 27, 28, 31, 33), and poor in one (26) (Table 1).

#### 3.2.1 Neonatal deaths

**Figure 5:**
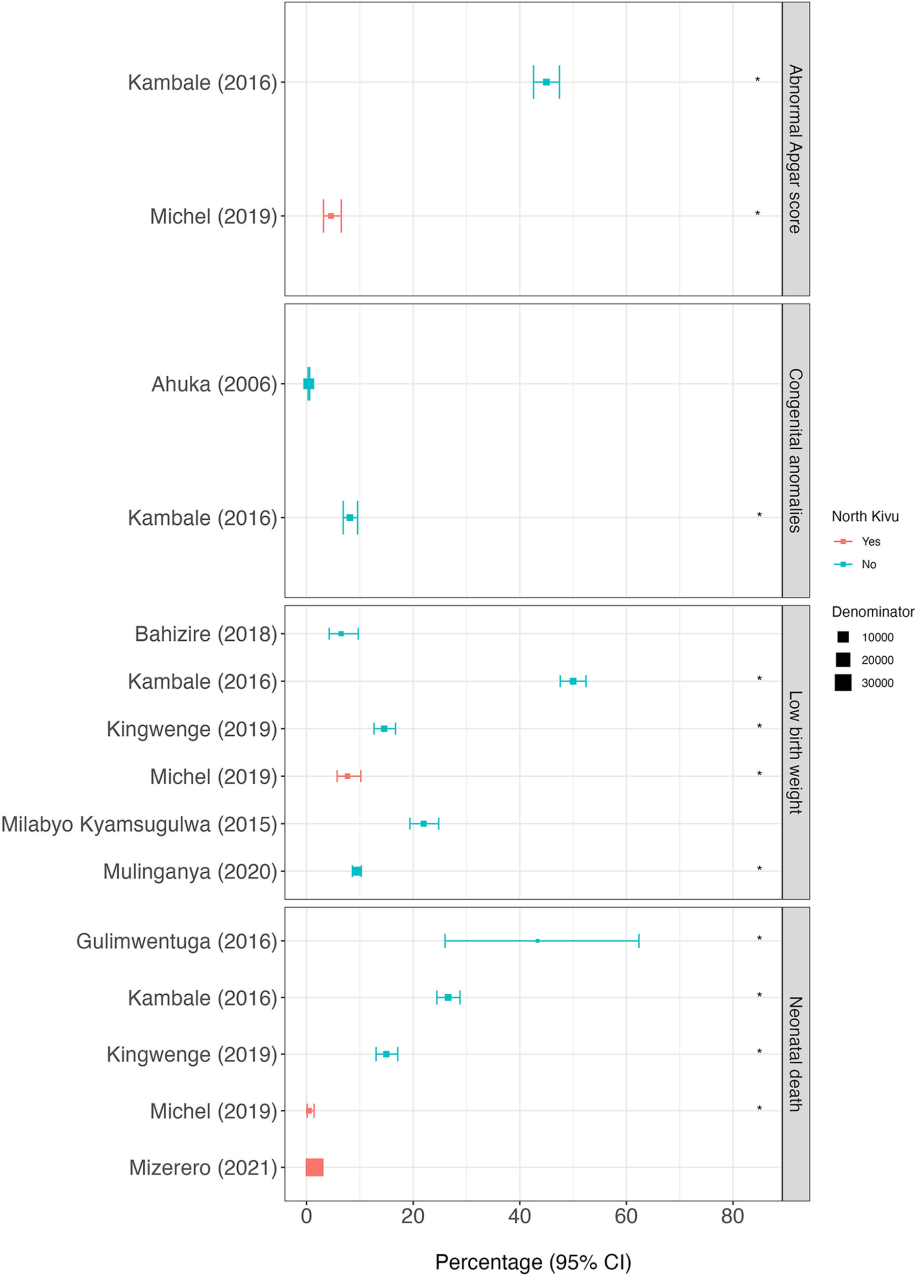
Proportion of neonatal outcomes for each scientific article. The position of squares represents the percentage of subjects with each outcome and the bars, the 95% confidence intervals (95%CI). The size of the squares is weighted accorded to the size of the denominator (provided in supplementary material 6). Red squares represent scientific articles conducted in North Kivu and blue squares, conducted outside North Kivu. The asterisk (*) indicates the scientific articles for which the denominator is non-standard. The abnormal Apgar score in Kambale (2016) is defined as “moderate asphyxia or state of apparent death” and in Michel (2019) as <7 at 10 min.

The percentages of neonates who died were obtained from five cross-sectional studies (25, 27, 28, 31, 33) and one retrospective cohort study (26), all of which only one (Mizerero et al., 2021) used a standard denominator (number of births) (Table 1 and Figure 5). In this study, conducted in 42 public health facilities in North Kivu, the percentage of very early (period undefined) neonatal deaths and intrapartum deaths among all births in those facilities was 1.5% (95%CI 1.4%–1.6%, *n* = 532/35,283) (33). In North Kivu, the percentage of neonates who died within 24 h and were born to women who had caesarean sections in four hospitals in Goma was 0.4% (95%CI 0.1%–1.4%, *n* = 3/676) (31) and 1.2% (95%CI 0.2%–4.6%, *n* = 2/173) of babies born to women prior to fistula surgery at a hospital in Goma died in less than a week after birth. In Maniema, 15.0% (95%CI 13.0%–17.1%, *n* = 184/1,230) of high risk infants admitted to the neonatal unit died (28) and in Bukavu, South Kivu, 43.3% (95%CI 26.0%–62.3%, *n* = 13/30) of infants aged 1–30 days admitted to a surgical emergency died (26).

An abnormal Apgar score was recorded in two scientific articles (27, 31), both of which used a non-standard denominator (Table 1 and Figure 5). In North Kivu, in a cross-sectional study of neonates born to women who had a caesarean section in four referral hospitals in Goma, an Apgar score of less than seven at 10 min was recorded in 4.6% (95%CI 3.2%–6.5%, *n* = 31/677) (31). In South Kivu, a cross-sectional study conducted in the neonatology ward of the provincial referral hospital of Bukavu reported an abnormal Apgar score, including moderate asphyxia or state of apparent death, in 45.0% (95%CI 42.6%–47.4%, *n* = 737/1,638) of hospitalised neonates (27) (Figure 5 and Supplementary Material S6).

#### 3.2.2 Low birth weight

The percentage of low birth weight (LBW) babies was obtained from five cross-sectional studies (27, 28, 31, 32, 35), and one prospective cohort study (24), of which four used a non-standard denominator (Table 1 and Figure 5). In North Kivu, in a cross-section study conducted in Goma, 7.7% (95%CI 5.7%–10.2%, *n* = 46/599) of babies born to mothers who had a caesarean section had LBW (31). In South Kivu, the percentage of LBW babies was 6.5% (95%CI 4.2%–9.7%, *n* = 23/355) of neonates born to women who attended an antenatal clinic in their second trimester in South Kivu (24). In Maniema, LBW infants were found in 22.0% (95%CI 19.4%–24.8%, *n* = 206/938) of live births in a maternity wards of two rural referral hospitals (32).

#### 3.2.3 Congenital anomalies

The percentage of infants with congenital anomalies was obtained from two cross-sectional studies, both conducted outside North Kivu (23, 27) of which one used a non-standard denominator (Table 1 and Figure 5). In Ituri, 0.4% (95%CI 0.3%–0.6%, *n* = 36/8,824) of all live deliveries in a referral hospital from 1993 to 2001 had a congenital anomaly, with the most commonly reported being clubbed foot (*n* = 9/8,824, 1 per 1,000 births), congenital hydrocephalus (*n* = 8/8,824, 0.9 per 1,000 births) and spina bifida (*n* = 6/8,824, 0.6 per 1,000 births) (23). In South Kivu, in the neonatology ward of Bukavu Tertiary Care Hospital, 8.1% (95%CI 6.9%–9.6%, *n* = 133/1,638) of neonates were reported to have a congenital anomaly (non-specified) (27).

## 4 Discussion

Despite the heterogeneity and varying quality of data collection methods in the studies included in the review, the findings suggest that this part of sub-saharan Africa has experienced a burden of adverse maternal, birth and neonatal outcomes that is comparable to the neighbouring African countries including those not currently experiencing armed conflict. This is further described below.

### 4.1 Pregnancy outcomes

Our review reported caesarean section rates ranging from 12 to 16% of all deliveries in the scientific articles analysed (31, 34, 35). However, evidence suggests that increasing caesarean sections rates beyond 10% does not confer additional benefits (41). Furthermore, in many resource-constrained settings, a significant proportion of maternal and newborn fatalities are directly related to caesarean sections performed under suboptimal conditions (42). In areas of conflict, a decline in caesarean section rates might be expected as women have less access to healthcare. However, the presence of humanitarian actors in these areas, particularly during protracted conflict, may actually increase the rates of caesarean sections, especially if these services are subsided (35). This could explain why the caesarean section rate in Eastern DRC is higher than the DRC national average of 5.1% reported between 2008 and 2014 (43). In neighbouring Uganda, while the overall population rate of caesarean sections was 5.9% in 2016, the rate among live births in health facilities was 11% (44), which is more comparable to the rates observed in our review of Eastern DRC. Similarly, in neighbouring Rwanda, the population-based caesarean section rate was 15.6% in 2019–2020, closely aligning with the rates observed in our review (45). However, both Uganda and Rwanda have very different socio-political and economic environments compared to Eastern DRC, with no active conflict and a much lower presence of humanitarian actors. Neighbouring Burundi, which is economically poorer and more fragile in terms of security than Uganda and Rwanda (46) is more similar to Eastern DRC. In Burundi, the estimates of caesarean section rates among all live births in 2016 varied from 7.1% to 15.3% depending on geographical location, wealth and education status of the women (46).

Pelvic anomalies were cited as the most common indication for caesarean sections in five tertiary care hospitals in South Kivu (29), and were the second most common indication in four referral hospitals in Goma (31). Cephalopelvic disproportion, which can result in obstructed labour, was the most frequently cited indication for caesarean sections in a multi-centre study in sub-Saharan Africa (47). A ’scarred uterus’, often found in women who have had a previous caesarean section, was the most common indication in one of the studies in Goma (31). This finding aligns with other publications both within and outside sub-Saharan Africa, where a previous caesarean section is often the most common indication for repeat procedures (48–53).

Regarding maternal mortality, the study by Mugisho et al. (2002) conducted in Rutshuru used a denominator that closely approximates the general population (women delivering in the maternity ward of the referral hospital, excluding refugees) (34). Since the data were from 1988 to 1998, comparing these figures with more recent data is challenging. The study reported a maternal mortality rate of 1.9% (34), which isnoticeably higher than the latest national estimate of 693 per 100, 000 live births (0.6%) in 2015 (54). However, this figure aligns more closely with the 1,188 deaths per 100, 000 live births (1.2%) reported in the DRC between 2012 and 2016 in a multi-country prospective cohort study (55), 1,300 per 100,000 live births (1.3%) in Maniema in 2020 (56) and 1,099 per 100,000 live births (1.1%) in Morogoro, Tanzania, in 1995 (57). The maternal mortality rate in the 2020 Maniema study (1.3%) was comparable to rates reported in other conflict affected regions, such as Central African Republic (835 per 100, 000 live births, 0.8%) in 2020 (58) and South Sudan (789 per 100, 000 live births, 0.8%) in 2015 (59). Despite using a non-standard denominator, the maternal mortality rate of 0.1% reported by Michel et al. (2019) in Goma among women who had a caesarean section (31) was much lower than the national estimate.

The heterogeneity of the denominators used in the scientific reviews for estimating miscarriage rates makes it challenging to determine the true magnitude of miscarriages in Eastern DRC. Consequently, estimates of miscarriages ranged from 7.3% to 30%. These figures are notably higher than the 2.5% incidence of miscarriage reported in a longitudinal study conducted in Tanzania, where 2.5% of 157 women attending antenatal clinics from their second trimester to term experienced a miscarriage between September 2008 and April 2009 (60).

Similarly, the variation in denominators used to estimate preterm birth rates in the scientific articles included in our review resulted in a wide range of estimates from 5.1% to 74%. These estimates do not align with rates reported in a population-based study conducted from 2014 to 2018 which found preterm birth rates of 18.3% in the DRC, 12.4% in Zambia and 9.8% in Kenya (61), as well as the 19.4% found in rural Uganda from 2014 to 2016 (62) and the 20.2% found in Nairobi, Kenya (63). However, the lower end of our estimates is more consistent with the 5.3% incidence from the Demographic and Health Surveys (DHS) from 2006 to 2018 across 36 Sub-Saharan African countries (64) and the 5% found in post-conflict northern Uganda from 2017 to 2019 (65). It is well recognised that preterm birth rates can vary between and within countries, often without a clear identification of the underlying causes of these disparities (66).

### 4.2 Neonatal outcomes

In the only scientific article which included a standard denominator in our review, the percentage of neonatal deaths among all live births of 2% (33), which is consistent with the national average of 3% (67). The neonatal death rate of1.5% reported by Mizerero et al.(2021) aligns with rates observed in studies from the region including Eastern Uganda in 2013 (3.0% to 3.6%) (68), Tanzania (2.4% in 2009 to 8.1% in 1985) (57), Kenya in 2018 (1.9%) (69), and Ethiopia from 2008 to 2013 (2.7%) (70). This is noteworthy given that neonatal mortality is generally higher in countries experiencing armed conflict and war (1). Similar to the findings on caesarean section rates, the longstanding presence of humanitarian actors in Eastern DRC may explain these relatively lower neonatal mortality rates.

The estimates for LBW in our review included two scientific articles with a standard denominator (all neonates), ranging from 6% to 22% (24, 32). The study by Bahizire et al.(2018), which reported a prevalence of 6.5% (24),, was more comparable with the national incidence of 10% in 2014 (67), the South Kivu incidence of 11% in 2013–2014 (71), and the 7.3% incidence in post-conflict northern Uganda from 2017 to 2019 (65). However, the lower estimates in Bahizire et al.(2018) compared to those reported by Milabyo Kyamusugulwa et al.(2006) could be attributed to the inclusion of only full-term neonates in the former study (24, 32). It is worth noting that global LBW rates have decreased over time (72). Despite evidence linking armed conflicts with higher LBW rates (73), our estimates, except for the study by Milabyo Kyamusughulwa et al. (2006), were inline or slightly lower than thise reported in DHS data from other sub-Saharan countries not experiencing insecurity, including Ghana (10.2%), Malawi (12.2%), and Senegal (15.7%) (74). Ahuka et al.(2006) reported a 1% incidence of congenital anomalies among all live births between 1993 and 2001 at a single hospital in Ituri (23). This is lower than the pooled prevalence of birth defects, which was 20.4 per 1,000 births (95%CI 17.0–23.7) from 25 studies in nine countries in sub-Saharan Africa (75). However, in resource-limited like Ituri, it is possible that some cases went undetected.

### 4.3 Study limitations

The heterogeneity in the proportion of pregnant women and their infants experiencing adverse outcomes is likely largely due to differences in study design, particularly the selection of the study populations. As a result, we did not provide pooled estimates for any of the outcomes. This heterogeneity also prevented us from conducting statistical analyses to compare the incidence of outcomes between North Kivu and other provinces.

## 5 Conclusion

This systematic review provides a comprehensive overview of pregnancy and neonatal outcomes over the past twenty years in Eastern DRC, highlighting the significant burden of adverse outcomes experienced by pregnant women and their infants. Despite the ongoing conflict, we found that pregnancy and neonatal outcomes in Eastern DRC are comparable to those in other countries in the region that are not currently experiencing armed conflict. Further studies aimed at understanding this resilience of the DRC health system in this war- affected area will be of great benefit.

## Data Availability

The original contributions presented in the study are included in the article/Supplementary Material, further inquiries can be directed to the corresponding author.

## Scope statement

This review examines research articles published between 2001 and 2021 across four provinces in eastern Democratic Republic of the Congo (DRC). It was originally initiated to support an Ebola vaccine trial conducted in Goma from 2019 to 2022, which included pregnant women. In the absence of a control arm, it was necessary to gather population-level data on the incidence of maternal and neonatal outcomes to contextualize the trial's safety data and potentially inform future studies. Given that eastern DRC has experienced prolonged armed conflict, obtaining unbiased estimates of pregnancy and neonatal outcomes has been challenging, reinforcing the need for a systematic review. Our review provides a comprehensive overview of pregnancy and neonatal outcomes over the past two decades, utilizing a range of studies. It highlights the substantial burden of adverse outcomes experienced by pregnant women and their babies; interestingly, these outcomes are comparable to those in neighboring countries not affected by conflict.
