# Supplementary material for: Pregnancy and neonatal outcomes in Eastern Democratic Republic of the Congo: a systematic review
Source: Front Glob Womens Health. 2024 Dec 5;5:1412403. doi: 10.3389/fgwh.2024.1412403 (PMC11655456; doi:10.3389/fgwh.2024.1412403)
Supplement: Supplementary file 1 [file Table1.docx]

**Supplementary material 1. Search strategy used for databases.**

| **Database(s)** | **Search strategy** | **Limits** |
| --- | --- | --- |
| Medline  EMBASE | (Pregnan* OR Matern* OR Mother OR F?etal OR F?etus OR (exp Pregnancy/) OR Neonat* OR Newborn OR Infant* OR Baby OR Babies OR (exp Infant, Newborn/) OR Deliver* OR Birth OR Born OR Outcome* OR Complication* OR Event*OR Miscarriage OR Abortion OR Preterm OR Prematur* OR Caesarean OR C-section OR Stillbirth OR Neonatal death OR Low birth weight OR Low birthweight OR Congenital anomal* OR Congenital abnormalit* OR (exp Fetal Death/) OR (exp Infant Death/) OR (exp Embryo Loss/) OR (exp Perinatal Death/) OR (exp Maternal Death/) OR (exp Congenital Abnormalities/))  AND (Kivu OR Ituri OR Eastern Democratic Republic of the Congo OR Eastern DR Congo OR Eastern DRC OR Goma OR Bukavu OR Butembo OR Beni OR Katwa OR Maniema OR Masisi OR Bunia OR Rutshuru) | Abstracts;  Human;  Date of publication: 2001-2021 |
| Global Health | (Pregnan* OR Matern* OR Mother OR F?etal OR F?etus OR (exp Pregnancy/) OR Neonat* OR Newborn OR Infant* OR Baby OR Babies OR (exp Infant, Newborn/) OR Deliver* OR Birth OR Born OR Outcome* OR Complication* OR Event*OR Miscarriage OR Abortion OR Preterm OR Prematur* OR Caesarean OR C-section OR Stillbirth OR Neonatal death OR Low birth weight OR Low birthweight OR Congenital anomal* OR Congenital abnormalit* OR (exp Fetal Death/) OR (exp Infant Death/) OR (exp Embryo Loss/) OR (exp Perinatal Death/) OR (exp Maternal Death/) OR (exp Congenital Abnormalities/))  AND (Kivu OR Ituri OR Eastern Democratic Republic of the Congo OR Eastern DR Congo OR Eastern DRC OR Goma OR Bukavu OR Butembo OR Beni OR Katwa OR Maniema OR Masisi OR Bunia OR Rutshuru) | Abstracts;  Date of publication: 2001-2021 |
| Cochrane | (Pregnan* OR Matern* OR Mother OR F?etal OR F?etus OR Neonat* OR Newborn OR Infant* OR Baby OR Babies OR Deliver* OR Birth OR Born OR Outcome* OR Complication* OR Event*OR Miscarriage OR Abortion OR Preterm OR Prematur* OR Caesarean OR C-section OR Stillbirth OR Neonatal death OR Low birth weight OR Low birthweight OR Congenital anomal* OR Congenital abnormalit*) AND (Kivu OR Ituri OR Eastern Democratic Republic of the Congo OR Eastern DR Congo OR Eastern DRC OR Goma OR Bukavu OR Butembo OR Beni OR Katwa OR Maniema OR Masisi OR Bunia OR Rutshuru) | Date of publication Jan 2001-May 2021 |
| ClinicalTrials.gov | Pregnancy \| Congo, The Democratic Republic of the  Neonatal\| Congo, The Democratic Republic of the |  |
