# Supplementary material for: Pregnancy and neonatal outcomes in Eastern Democratic Republic of the Congo: a systematic review
Source: Front Glob Womens Health. 2024 Dec 5;5:1412403. doi: 10.3389/fgwh.2024.1412403 (PMC11655456; doi:10.3389/fgwh.2024.1412403)
Supplement: Supplementary file 2 [file Table2.docx]

**Supplementary material 2.** **Search strategy used for grey literature.**

| **Database(s)** | **Search strategy: English** | **Search strategy: French** | **Filters applied** |
| --- | --- | --- | --- |
| Google | Pregnancy outcomes OR neonatal outcomes Kivu OR Ituri OR Eastern  Democratic Republic of the Congo OR Eastern DR Congo OR Eastern DRC OR Goma OR  Bukavu OR Butembo OR Beni OR Katwa OR Maniema OR Masisi OR Bunia OR Rutshuru | Issues de la grossesse OR issues néonatales Kivu OR Ituri OR Est  République démocratique du Congo OR Est de la RDC OR Est de la RDC OR Goma OR  Bukavu OR Butembo OR Beni OR Katwa OR Maniema OR Masisi OR Bunia OR Rutshuru | NA |
| The Demographic Health Survey Program | NA | NA | Filters applied: DRC, years 2001-2021, language English and French |
| ReliefWeb | Pregnancy outcomes OR neonatal outcomes Kivu OR Ituri OR Eastern  Democratic Republic of the Congo OR Eastern DR Congo OR Eastern DRC OR Goma OR  Bukavu OR Butembo OR Beni OR Katwa OR Maniema OR Masisi OR Bunia OR Rutshuru | Issues de la grossesse OR issues néonatales Kivu OR Ituri OR Est  République démocratique du Congo OR Est de la RDC OR Est de la RDC OR Goma OR  Bukavu OR Butembo OR Beni OR Katwa OR Maniema OR Masisi OR Bunia OR Rutshuru |  |
| UNICEF | NA | NA | Pregnancy– limit DRC  Pregnancies – limit DRC  Neonatal – limit DRC  Newborn – limit DRC |
| MSF | Pregnancy Democratic Republic of the Congo  Neonatal Democratic Republic of the Congo | Grossesse République démocratique du Congo  Issues Néonatale République démocratique du Congo |  |
| USAID | Pregnancy Democratic Republic of the Congo  Neonatal Democratic Republic of the Congo | Grossesse République démocratique du Congo  Issues Néonatale République démocratique du Congo |  |
