# Supplementary material for: Pregnancy and neonatal outcomes in Eastern Democratic Republic of the Congo: a systematic review
Source: Front Glob Womens Health. 2024 Dec 5;5:1412403. doi: 10.3389/fgwh.2024.1412403 (PMC11655456; doi:10.3389/fgwh.2024.1412403)
Supplement: Supplementary file 3 [file Table3.docx]

**Supplementary material 3.** **Checklist for elimination of articles via screening of the abstracts.**

|  | **Questions** | **Yes** | **No** | **Unclear** |
| --- | --- | --- | --- | --- |
| 1. | Does the article describe one or more pregnancy outcomes? |  |  |  |
| 2. | Does the article describe one or more infant outcomes? |  |  |  |
| 3. | Is the data from the DRC? |  |  |  |
| 4. | Was the study (or report) published between 2001 and 2021? |  |  |  |
| 5. | Was the article published in a peer-reviewed journal (excluding grey literature)? |  |  |  |
| 6. | If the article was from grey literature was it published by the government or a listed NGO? |  |  |  |
| 7. | Is the article in either English or French? |  |  |  |
| 8. | Is the article an observational or interventional study (and not a case report or case series) *Scientific articles only* |  |  |  |
|  | *If the answer to any of the questions is ‘No’ the study is not eligible.*  *If any answers are ‘Unclear’ the full-text must be retrieved for clarity* |  |  |  |
