# Supplementary material for: Pregnancy and neonatal outcomes in Eastern Democratic Republic of the Congo: a systematic review
Source: Front Glob Womens Health. 2024 Dec 5;5:1412403. doi: 10.3389/fgwh.2024.1412403 (PMC11655456; doi:10.3389/fgwh.2024.1412403)
Supplement: Supplementary file 4 [file Table4.docx]

**Supplementary material 4.** **Excluded scientific articles, reports, and articles.**

| **Number** | **First author, year** | **Region** | **Study design** | **Time of data collection** | **Outcomes** | **Reason for exclusion** |
| --- | --- | --- | --- | --- | --- | --- |
| 1 | Ahuka, 2004 | Niakunde, Ituri | Cross-sectional | 1993-1997 and 1997-2001 | Miscarriage  Stillbirth  Preterm birth | Numerators and denominators not provided |
| 2 | NICHD Global Network for Women's and Children's Health, 2010 | Unclear | Prospective cohort | 2008-2023 | Congenital anomaly  IUGR  Low birth weight  Maternal death  Miscarriage  Neonatal death  Stillbirth | Absence of data specifically for eastern DRC |
| 3 | NICHD Global Network for Women's and Children's Health, 2015 | Unclear | Randomised clinical trial | 2016-2018 | Low birth weight  Maternal anaemia  Maternal death  Miscarriage  Neonatal mortality  Preterm birth  Pre-eclampsia  Small for gestational age  Stillbirth  Postpartum haemorrhage | Absence of data specifically for eastern DRC |
| 4 | Bahizire, 2017 | Miti-Murhesa, South Kivu | Cross-sectional | 2013-2014 | Maternal anaemia | Numerators and denominators not provided |
| 5 | Cikomola, 2017 | Walungu health zone, South Kivu | Cross-sectional | 2015-2016 | Neonatal death | Numerators and denominators not provided |
| 6 | DHS, 2007 | National | NA | 2007 | Maternal death | Absence of data specifically for eastern DRC |
| 7 | DHS II, 2007 | National | NA | 2007 | Maternal death  Caesarean section  Low birth weight  Neonatal death | Numerators and denominators not provided |
| 8 | DHS, 2013-2014 | National | NA | 2013-2014 | Maternal anaemia  Low birth weight | Numerators and denominators not provided |
| 9 | DHS II, 2013-2014 | National | NA | 2013-2014 | Maternal anaemia | Numerators and denominators not provided |
| 10 | EPSS, 2017-2018 | National | NA | 2017-2018 | Nil | Absence of data on relevant outcomes |
| 11 | Kabuiku, 2018 | Lubumbashi | Cross-sectional | 2007 | Post-partum haemorrhage | Conducted outside study catchment area |
| 12 | Kapteni, 2018 | North Kivu | Cross-sectional | 2017 | Causes of maternal deaths | Absence of data on relevant outcomes |
| 13 | Kimona, 2012 | Goma, North Kivu | Cross sectional | 2011 | Nil | Absence of data on relevant outcomes |
| 14 | Longombe, 2012 | Goma, North Kivu | Case series | 2002-2004 | Congenital anomalies | Case series |
| 15 | Malemo Kaliysa, 2015 | Goma, North Kivu | Case series | 2002-2014 | Congenital anomalies | Case series |
| 16 | Maleya, 2019 | Lubumbashi | Cross-sectional | 2013-2014 | Eclampsia  Maternal anaemia  IUGR  Caesarean section  Preterm birth  Prelabour rupture of membranes  Maternal death  Low birth weight  Neonatal death  Apgar score  Prolonged hospitalisation | Conducted outside study catchment area |
| 17 | Maroyi, 2018 | South Kivu | Cross-sectional | Unknown | Caesarean sections | Numerators and denominators not provided |
| 18 | MICS – Palu, 2019 | National | NA | 2017-2018 | Nil | Absence of data on relevant outcomes |
| 19 | Mugisho, 2002 (Newborn deaths) | Rutshuru, North Kivu | Cross-sectional | 1980-1998 | Low birth weight  Neonatal death | Numerators and denominators not provided |
| 20 | Mugisho, 2003 | Kirotshe and Rutshuru, North Kivu | Cohort | 1995-1996 | Maternal death  Caesarean section  Low birth weight  Neonatal death | Numerators and denominators not provided |
| 21 | Mullen, 2005 | National | NA | 2005 | Nil | Absence of data on relevant outcomes |
| 22 | Mulyumba | Goma, North Kivu | Case control | 2008-2013 | Nil | Absence of data on relevant outcomes provided |
| 23 | Pau, 2014 | Lake Kivu | NA | Unknown | Maternal death  Neonatal death | Absence of data specifically for eastern DRC |
| 24 | Planification familiale, 2013 | National | NA | 2013 | Nil | Absence of data on relevant outcomes |
| 25 | Project Concern USAID, 2015 | National | NA | Unknown | Caesarean section  Preterm birth  Maternal death  Low birth weight  Neonatal death | Absence of data specifically for eastern DRC |
| 26 | Rouhani, 2016 | Bukavu, South Kivu | Mixed methods | 2012 | Nil | Absence of data on relevant outcomes |
| 27 | WHO, 2012 | National | NA | 1990-2010 | Maternal death | Absence of data specifically for eastern DRC |
| 28 | Patrick Mullen,2005 | National | NA | 2015 | Nil | Absence of data on relevant outcomes (Duplicate of Mullen 2005) |
| 29 | Lututala Mumpasi Bernard,2007 | National | NA | 2007 | Maternal death  Caesarean section  Low birth weight  Neonatal death | Numerators and denominators not provided.  (Duplicate of DHS II, 2007) |
