# Supplementary material for: Pregnancy and neonatal outcomes in Eastern Democratic Republic of the Congo: a systematic review
Source: Front Glob Womens Health. 2024 Dec 5;5:1412403. doi: 10.3389/fgwh.2024.1412403 (PMC11655456; doi:10.3389/fgwh.2024.1412403)
Supplement: Supplementary file 5 [file Table5.docx]

**Supplementary material 5.** **Data extraction form.**

| **Data** | **Comments/results** |
| --- | --- |
| Title |  |
| First author |  |
| Year of publication |  |
| Year of study |  |
| Type of study |  |
| Study objectives |  |
| Setting |  |
| Study population |  |
| Dates of data collection |  |
| Data source |  |
| Sample size |  |
| Results (n (%, 95%CI)*  ***During pregnancy***  1. Miscarriage  2. Pre-eclampsia  3. Eclampsia  4. Gestational diabetes  5. Hyperemesis gravidarum  6. Anaemia  7. Intrauterine growth restriction (IUGR)  8. Placenta praevia  9. Maternal death  10. Stillbirth  ***Labour***  1. Caesarean section  2. Reasons for Caesarean section  3. Preterm birth  4. Prelabour rupture of membranes  5. Post-partum haemorrhage  6. Maternal death  ***Infant***  1. Low birth weight  2. Neonatal death  3. Congenital anomaly  4. Apgar score  5. Small for gestational age  6. Prolonged hospitalisation |  |
| Study limitations including biases |  |
