# Supplementary material for: Pregnancy and neonatal outcomes in Eastern Democratic Republic of the Congo: a systematic review
Source: Front Glob Womens Health. 2024 Dec 5;5:1412403. doi: 10.3389/fgwh.2024.1412403 (PMC11655456; doi:10.3389/fgwh.2024.1412403)
Supplement: Supplementary file 6 [file Table6.docx]

**Supplementary material 6.** **Definition of outcomes and corresponding denominators by scientific article**

| **First author, year (reference)** | **Outcome** | **Definition of the outcome** | **Denominator** |
| --- | --- | --- | --- |
| Ahuka, 2006 (26) | Congenital anomalies | Congenital anomalies clinically present at birth or on examination at hospital admission using Wacker’s definition of birth defect | Babies delivered alive (n=8824) |
| Bahizire, 2018 (27) | Maternal anaemia | Haemoglobin of <110g/L | Pregnant women in the second trimester for whom a haemoglobin level was obtained at enrolment at the first ANV (n=439) |
| Bahizire, 2018 (27) | Low birthweight | Birthweight <2500g | Live births amongst pregnant women recruited during their second trimester of pregnancy (n=355). |
| Benfield, 2015 (28) | Caesarean section | Not applicable | Pregnant women who underwent fistula surgery for whom data on the mode of delivery were available (n=176) |
| Benfield, 2015 (28) | Neonatal death | Died within 1 week of birth | Pregnant women who underwent fistula surgery for whom data on the neonatal outcome was available (n=173) |
| Benfield, 2015 (28) | Stillbirth | Not provided* | Pregnant women who underwent fistula surgery for whom data on the neonatal outcome was available (n=173) |
| Gulimwentuga, 2016 (29) | Neonatal death | Deaths in neonates aged 1-30 days | Neonates aged 1-30 days who were hospitalised for a surgical emergency (n=30) |
| Gulimwentuga, 2016 (29) | Preterm birth | Not provided* |  |
| Kambale, 2016 (30) | Apgar score | Not provided* | Hospitalised neonates aged 0 to 28 days with complete medical records **(n=1638) |
| Kambale, 2016 (30) | Congenital anomalies | Not provided* |  |
| Kambale, 2016 (30) | Low birth weight | Birthweight <2500g |  |
| Kambale, 2016 (30) | Neonatal death | Death of a neonate aged 0 to 28 days |  |
| Kambale, 2016 (30) | Preterm birth | Not provided * |  |
| Kingwenge, 2019 (31) | Low birth weight | Not provided* | New-borns admitted to the neonatal unit of Kindu General Reference Hospital in Maniema Province. |
| Kingwenge, 2019 (31) | Neonatal death | Not provided* |  |
| Kingwenge, 2019 (31) | Preterm birth | Not provided* |  |
| Maroyi, 2020 (32) | Caesarean section | Planned or emergency caesarean section | All women who had a delivery (vaginally or via planned or emergency caesarean section) with a history of two or more previous caesarean sections at one of the five tertiary care hospitals in South Kivu (n=422). Of the women, 36% (n=151) delivered at Panzi, 21% (n=88) delivered at Rau, 19% (n=79) at Ifendula, 12% (n=52) at Kalonge and 12% (n=52) at Nyantende. |
| Maroyi, 2020 (32) | Reason for caesarean section | The reasons for caesarean section included: contracted pelvis, haemorrhage, uterine rupture, abnormal foetal presentation, and foetal distress. |  |
| Mbusa-Kambale, 2018 (33) | Intrauterine growth restriction | Birth weight below the 3^rd^ percentile of the AUDIPOG’s reference of birth weight-for-gestational-age† | 100 new-borns with a birth weight of <2500g and 100 new-borns with a birth weight of $\geq$2500g who were born full term in the neonatology units and the maternal and child health unit of Reference Provincial General Hospital of Bukavu, South Kivu |
| Mbusa-Kambale, 2018 (33) | Preterm birth | Gestational age at delivery <37 weeks no | 100 new-borns with a birth weight of <2500g who were born in the neonatology unit and the maternal and child health unit of Reference Provincial General Hospital of Bukavu, South Kivu |
| Michel, 2019 (34) | Apgar score | Not provided* | Number of women who had a caesarean section and for whom the Apgar score was available for the neonate at one of four referral hospitals in Goma (Bethesda Hospital, Virunga Hospital, Charité Maternelle Hospital and Goma Provincial Referral Hospital), North Kivu between November 2013 and January 2016 |
| Michel, 2019 (34) | Caesarean section | N/A | Number of deliveries at one of four referral hospitals (Bethesda Hospital, Virunga Hospital, Charité Maternelle Hospital and Goma Provincial Referral Hospital), Goma, North Kivu between November 2013 and January 2016 |
| Michel, 2019 (34) | Reasons for caesarean section | Post-operative reasons for caesarean section including:  Pelvic anomaly, scarred uterus, dynamic dystocia, foetal distress, placenta praevia, other reasons. | Number of women who had a caesarean section and for whom data were available on the indication for caesarean section at one of four referral hospitals, (Bethesda Hospital, Virunga Hospital, Charité Maternelle Hospital and Goma Provincial Referral First Hospital), in Goma, North Kivu between November 2013 and January 2016 |
| Michel, 2019 (34) | Low birth weight | <2500g | Number of women who had a caesarean section and for whom data were available on the birth weight of the neonate at one of four referral hospitals (Bethesda Hospital, Virunga Hospital, Charité Maternelle Hospital and Goma Provincial Referral Hospital), in Goma, North Kivu between November 2013 and January 2016 |
| Michel, 2019 (34) | Maternal death | Not provided* |  |
| Michel, 2019 (34) | Miscarriage | Not provided* |  |
| Michel, 2019 (34) | Neonatal death | Death within 24 hours of birth |  |
| Michel, 2019 (34) | Placenta praevia | Post operative diagnosis of placenta praevia |  |
| Michel, 2019 (34) | Stillbirth | Fresh, macerated, and unspecified |  |
| Milabyo Kyamusugulwa, 2006 (35) | Low birth weight | <2500g | Infants born at the maternity ward of the Kama Referral Health Center and the maternity ward of the Kipaka General Referral Hospital in Maniema during the study period |
| Mizerero, 2021 (36) | Caesarean section | Not applicable | Number of births in 42 public health facilities providing maternal and neonatal health services in Goma, Karisimbi, and Rutshuru Health Zones in North Kivu in 2017 |
| Mizerero, 2021 (36) | Neonatal death | Very early (period not defined) and intrapartum death |  |
| Mizerero, 2021 (36) | Maternal death | Not provided* | Number of major direct obstetric complications in 42 public health facilities providing maternal and neonatal health services in Goma, Karisimbi, and Rutshuru Health Zones in North Kivu in 2017 |
| Mizerero, 2021 (36) | Miscarriage | Not provided* |  |
| Mizerero, 2021 (36) | Pre-eclampsia | Not provided* |  |
| Mugisho, 2002 (Maternal deaths) (37) | Caesarean section | Not provided* | Women delivering in the maternity ward of Rutshuru Referral Hospital in North Kivu between 1980 and 1998 (excluding refugees) |
| Mugisho, 2002 (Maternal deaths) (37) | Maternal deaths | Not provided* |  |
| Mulinganya, 2020 (38) | Caesarean section | Not applicable | Number of deliveries at 30 secondary health facilities in eight Health Zones in South Kivu between January and December 2018 |
| Mulinganya, 2020 (38) | Low birth weight | <2500g | Number of caesarean Sections at 30 secondary health facilities in eight Health Zones in South Kivu between January and December 2018 |
| Mulinganya, 2020 (38) | Miscarriage | Not provided* |  |
| Mulinganya, 2020 (38) | Preterm birth | Not provided* |  |
| Richard, 2020 (39) | Miscarriage | Not provided* | Cases: pregnant women diagnosed or followed up for pre-eclampsia in one of seven hospitals selected in Goma, North Kivu  Controls: women attending ante natal care visits with normal pregnancy and no apparent chronic or debilitating conditions in the same facility as the case in Goma, North Kivu |

*The definitions of outcomes were not provided in the authors ‘manuscript. We assumed that the authors referred to the standard definitions. For reference 17, placenta praevia was classified under pre-operative or post-operative indications for caesarean section.
For stillbirth, we assumed the cut-off of 28 weeks used by the World Health Organization (WHO) for international comparisons (stillbirth being a baby who dies after 28 weeks of pregnancy, but before or during birth).

** Complete medical records referred to medical records having all the variables analysed by the author.

†The authors used the AUDIPOG (Association des Utilisateurs de Données Informatisées en Pédiatrie, Obstétrique, et Gynécologie

) study reference curves for birth weight
