# Supplementary material for: Pregnancy and neonatal outcomes in Eastern Democratic Republic of the Congo: a systematic review
Source: Front Glob Womens Health. 2024 Dec 5;5:1412403. doi: 10.3389/fgwh.2024.1412403 (PMC11655456; doi:10.3389/fgwh.2024.1412403)
Supplement: Supplementary file 7 [file Table7.docx]

**Supplementary material 7.** **Corresponding data for the forest plots showing the incidence of pregnancy and neonatal outcomes (Figures 3 and 5).**

| **Author (year of publication)** | **Study conducted in North Kivu** | **Outcome** | **Numerator** | **Denominator** | **Percentage**  **(95% CI)** |
| --- | --- | --- | --- | --- | --- |
| Ahuka (2006) | No | Congenital anomalies | 36 | 8824 | 0.41  (0.29-0.57) |
| Bahizire (2018) | No | Low birth weight | 23 | 355 | 6.48  (4.24-9.70) |
| Bahizire (2018) | No | Maternal anaemia | 141 | 439 | 32.12  (27.81-36.74) |
| Benfield (2015) | Yes | Caesarean section | 79 | 176 | 44.89 (37.45-52.55) |
| Benfield (2015) | Yes | Neonatal death | 2 | 173 | 1.16 (0.20-4.55) |
| Benfield (2015) | Yes | Stillbirth | 128 | 173 | 73.99 (66.68-80.21) |
| Gulimwentuga (2016) | No | Neonatal death | 13 | 30 | 43.33  (25.98-62.34) |
| Gulimwentuga (2016) | No | Preterm birth | 8 | 30 | 26.67  (12.98-46.17) |
| Kambale (2016) | No | Abnormal Apgar score | 737 | 1638 | 44.99  (42.57-47.44) |
| Kambale (2016) | No | Congenital anomalies | 133 | 1638 | 8.12  (6.87-9.58) |
| Kambale (2016) | No | Low birth weight | 819 | 1638 | 50.00  (47.58-52.42) |
| Kambale (2016) | No | Neonatal death | 435 | 1638 | 26.56  (24.45-28.78) |
| Kambale (2016) | No | Preterm birth | 667 | 1638 | 40.72  (38.34-43.15) |
| Kingwenge (2019) | No | Low birth weight | 179 | 1230 | 14.55  (12.65-16.68) |
| Kingwenge (2019) | No | Neonatal death | 184 | 1230 | 14.96  (13.04-17.10) |
| Kingwenge (2019) | No | Preterm birth | 179 | 1230 | 14.55  (12.65-16.68) |
| Maroyi (2020) | No | Caesarean section | 398 | 422 | 94.31  (91.54-96.25) |
| Mbusa-Kambale (2018) | No | IUGR | 33 | 200 | 16.50  (11.78-22.54) |
| Mbusa-Kambale (2018) | No | Preterm birth | 74 | 100 | 74.00  (64.10-82.03) |
| Michel (2019) | Yes | Abnormal Apgar score | 31 | 677 | 4.58  (3.18-6.51) |
| Michel (2019) | Yes | Caesarean section | 736 | 4530 | 16.25  (15.19-17.36) |
| Michel (2019) | Yes | Maternal death | 1 | 676 | 0.15  (0.01-0.96) |
| Michel (2019) | Yes | Miscarriage | 91 | 658 | 13.83  (11.33-16.76) |
| Michel (2019) | Yes | Low birth weight | 46 | 599 | 7.68  (5.73-10.19) |
| Michel (2019) | Yes | Neonatal death | 3 | 676 | 0.44  (0.11-1.40) |
| Michel (2019) | Yes | Stillbirth | 30 | 676 | 4.44  (3.06-6.35) |
| Milabyo Kyamusugulwa (2015) | No | Low birth weight | 206 | 938 | 21.96  (19.38-24.78) |
| Mizerero (2021) | Yes | Caesarean section | 5134 | 44042 | 11.66  (11.36-11.96) |
| Mizerero (2021) | Yes | Maternal death | 28 | 545 | 5.14  (3.50-7.43) |
| Mizerero (2021) | Yes | Miscarriage | 40 | 545 | 7.34  (5.36-9.94) |
| Mizerero (2021) | Yes | Pre-eclampsia | 95 | 545 | 17.43  (14.39-20.94) |
| Mizerero (2021) | Yes | Neonatal death | 532 | 35283 | 1.51  (1.38-1.64) |
| Mugisho (2002) | Yes | Caesarean section | 1951 | 13042 | 14.96  (14.35-15.59) |
| Mugisho (2002) | Yes | Maternal death | 243 | 13042 | 1.86  (1.64-2.11) |
| Mulinganya (2020) | No | Caesarean section | 5520 | 29600 | 18.65  (18.21-19.10) |
| Mulinganya (2020) | No | Miscarriage | 578 | 3420 | 16.90  (15.67-18.21) |
| Mulinganya (2020) | No | Low birth weight | 446 | 4748 | 9.39  (8.59-10.27) |
| Mulinganya (2020) | No | Preterm birth | 168 | 3273 | 5.13  (4.41-5.95) |
| Richard (2020) | Yes | Miscarriage | 57 | 190 | 30.00  (23.69-37.13) |
| Richard (2020) | Yes | Pre-eclampsia | 21 | 190 | 11.05  (7.13-16.61) |
|  |  |  |  |  |  |
|  |  |  |  |  |  |
|  |  |  |  |  |  |
|  |  |  |  |  |  |
|  |  |  |  |  |  |
|  |  |  |  |  |  |
|  |  |  |  |  |  |
|  |  |  |  |  |  |
|  |  |  |  |  |  |
|  |  |  |  |  |  |
|  |  |  |  |  |  |
|  |  |  |  |  |  |
|  |  |  |  |  |  |
|  |  |  |  |  |  |
|  |  |  |  |  |  |
|  |  |  |  |  |  |
